# Progress in Medicine

**Published:** 1894-10-13

**Authors:** 


### Progress in Medicine.

ANÆSTHETICS—*(Continued)*.

**Ether.**—There are not the same differences of opinion as to the action and method of administration of this anæsthetic as there are in the case of chloroform. All are agreed that it makes the greatest demands upon the organs of respiration, and that owing to its stimulating action on the heart the initial cardiac syncope which is said sometimes to occur with chloroform narcosis is extremely unlikely to supervene. At the Berlin Medical Society Dr. Vogel[18] describes the two methods of etherisation practised, the one "rapidly by the administration of a massive dose and the other progressively in small doses. The former method quickly determines anæsthesia, which, however, is preceded by a period of rather violent excitation accompanied by cyanosis, dyspnœa, &c. By the second method anæsthesia is produced more slowly, although it does not require more than ten or twelve minutes on an average; moreover, the period of excitation is almost completely suppressed."

Many anæsthetists prefer commencing ether narcosis with the preliminary administration of nitrous oxide gas. The *Lancet*, February 10th, describes a new apparatus for this purpose invented by Mr. Rumboll. He claims for it the following advantages: (1) It is extremely simple and portable; (2) the body being of glass, the amount of ether used is always visible; (3) by giving gas first the struggling stage of anæsthesia is done away with, and the nauseous odour of ether is not perceived by the patient. The patient is got under in a very short time, first of all being allowed to breathe air, then gas until sufficiently insensible to be unable to smell; ether is then turned on, and anæsthesia continued with a mixture of gas and ether until complete insensibility is produced, when the gas is shut off and ether continued alone. The apparatus may be used for ether only or gas only.

There is a growing tendency, both in this country and America, and also on the Continent, to make use of ether as the routine anæsthetic, substituting chloroform when the former is contraindicated. Dr. Fowler[19] says that in America the question as to the relative merits of the two anæsthetics has been pretty generally settled in favour of ether. Körte,[20] after discussing the two, decides in favour of ether. He states that he gets less vomiting with it than with chloroform. Gane,[21] in a book on ether narcosis, says that vomiting occurs in about 40 per cent. of the cases either during or after the operation. He recommends for the prevention of this the subcutaneous injection of morphia and atropine previous to etherisation; also to diminish the struggling stage the injection of morphia is useful. He strongly advocates the use of ether, but says it is contraindicated in bronchitis, pneumonia, emphysema, compression of the trachea, and in operations on the neck and face. Hare and Thornton[22] consider chloroform not so safe as ether, but that it may be given with advantage in the following cases: "In hot climates when a large number of persons are to be rapidly anæsthetised, in Bright's disease, in aneurism or great atheroma of blood vessels, in children or adults who already have bronchitis, to persons who struggle violently."

It is essential, as in the case of chloroform, that the ether should be pure. Nogel[23] says: "In order to ensure the best results, it is of importance that the ether contain neither acetic nor sulphurous acid, and it is therefore necessary to keep it in a dark place, inasmuch as under the influence of light a certain quantity of acetic acid is generated."

With regard to the comparative safety of chloroform and ether, as shown by statistics, Guilt,[24] from a large number of cases, finds one death in 2,907 of chloroform, and one in 14,646 cases of ether narcosis. Hewett, in his book, refers to statistics collected by Ormsby and Julliard, from which in over a million administrations ether was found five times safer than chloroform, thus agreeing almost exactly with those of Guilt. A writer in the *Medical Week*, May 4th, however, who includes Guilt's statistics in his own, finds only one death in 26,000 etherisations, as against one in 1,924 chloroformisations, thus making ether almost fourteen times as safe as chloroform.

Ischmarke[25] relates a death from ether, the patient three times previously having taken this anæsthetic with impunity; the operation was to open an abscess in the abdominal wall. The first indication of danger was that the patient became blue, he then vomited and became more cyanosed. It was difficult to get the vomited matter out of the mouth. Artificial respiration was performed, but no air could be pressed out of the chest. A low tracheotomy was done and vomited matter sponged out of the trachea. The patient died, and at the autopsy some small masses of vomit were found in a very few medium-sized tubes, but no real obstruction could have been caused by them. Death occurred from asphyxia, due either to paralysis of the respiratory centres or to aspiration of the vomit into the lungs; the author thinks that the former was the cause of death.

**Nitrous Oxide.**—Adams[26] describes at length a death under nitrous oxide. A young man, about twenty-six years old, with a receding jaw and short thick neck, was to have a molar tooth out. After taking three or four inspirations of the gas he took off the face piece, but then allowed the administration to be continued; his respiration was shallow but regular, and after taking about two-thirds of the ordinary quantity of gas the tooth was extracted; the respirations at once became irregular and the patient became more cyanosed, his muscles rigid, and after three or four respirations he ceased to breathe. Artificial respiration was performed, the heart continuing to beat regularly but not strongly. Nitrite of amyl was applied to the nose and mouth, but as no inspirations took place it could not have affected the patient. A subcutaneous injection of ether was given over the precordium, but all these measures failing tracheotomy was performed, and through the wound made a quantity of mucus was forced out by artificial respiration. No voluntary effort at breathing was made from first to last. The autopsy showed venous congestion of the internal organs, the heart, with the exception of a little fluid blood in the right ventricle, being empty. Mr. Adams attributes the patient's death to asphyxia. Sir G. Johnson[27] in some remarks on the case suggests that the cause of death should be termed apnœa rather than asphyxia. He also suggests that a drop or two of nitrite of amyl injected under the skin might be of service, also that venesection might be resorted to in similar circumstances. Dr. Hewitt[28] considers that with few exceptions, in about 90 per cent. of all cases oxygen should be given with the nitrous oxide, thus depriving it of its one drawback, viz., its asphyxiating properties. Where thus administered the swelling of the tongue, tonsils, and adjacent parts, the obstructive stertor, and the small, quick radial pulse cease to manifest themselves. Thomburg[29] relates the death of a woman after inhaling four gallons of nitrous oxide gas. The patient first showed signs of embarrassed breathing, the pulse became rapid, and attempts at breathing spasmodic. Artificial respiration was performed, nitro glycerine ($\frac{1}{100}$ grain) injected hypodermically, and ammonia applied to the nostrils. The patient seemed to rally for a short time, but finally died. The autopsy disclosed congested and œdematous lungs, with frothy exudate in bronchi, and nothing else worthy of note.

**Bromide of Ethyl.**—M. Eloy,[30] in describing the chemical characteristics of this drug says: "It is a colourless liquid, refracting and dense, the odour is like that of chloroform; it has a burning taste, and boils at 100·2 deg. Fahr., or 102·1 deg. It is insoluble in water, soluble in alcohol, in ether, in chloroform, and in oil; it is slightly inflammable, and as it decomposes on exposure to air, light, or damp, it should be kept in yellow bottles carefully corked." Continuing, he says, "it produces narcosis in two stages. The first is that of partial anæsthesia with full consciousness; the sensibility and the perceptions become deadened, and there is little or no trouble with the respiration or the circulation. In the second stage there is anæthesia with loss of consciousness, trembling of the muscles, and dilatation and insensibility of the pupils. . . . Its action is powerful, and above all rapid. Narcosis follows its administration in from thirty seconds to a minute, and is kept up by inhalations repeated every minute for about ten or fifteen minutes, beyond which time it is dangerous to continue the inhalations. . . . On the patient's awakening there is a prompt return to the normal condition, and there is very rarely any vomiting, headache, or contraction of the pupils. . . . It cannot be used in long operations or where the loss of blood is likely to be considerable, as it may increase it. The clinical contraindications, according to Stemfeld, are pulmonary tuberculosis, acute and chron c bronchitis, pulmonary emphysema, cardiac disease, pronounced anæmia, and, according to Gunsburg, alcoholism. . . It may be used before chloroform inhalations as a sort of preparatory anæsthesia to shorten the initial stage of chloroform narcosis. . . The following method of using ethyl-bromide is recommended: "A flannel mask which has been thoroughly saturated with the drug is applied quickly to the nose and the mouth in order to keep out the air. The face becomes discoloured, ocular congestion and muscular relaxation follow, and at this moment the operation should be performed." In "mixed" anæsthesia, that is to say when chloroform is to follow the ethyl-bromide, Chabouse gives the following rules: "(1) Cover the face as far as the chin with a flannel mask having a wire framework. (2) Thoroughly saturate the flannel with from 150 to 225 grains of ethyl-bromide, and repeat this in twenty seconds with the same quantity. (3) Keep the mask on for forty or forty-five seconds, when slight suffocation, facial congestion, and swelling of the vessels of the neck will take place. (4) Replace the ethyl-bromide with chloroform, which is administered by the standard method."

Professor Pavlow,[31] after describing the chemical characters of ethyl-bromide, points out that it must not be confounded with bromide of ethylene, which is a violent poison. He advises the bromethyl-chloroform mixture, the patient being anæsthetised with the former substance, and the anæsthesia being subsequently kept up by the latter. Having reviewed the subject, the draws up the following reasons why the above-mentioned mixture should be preferred to pure chloroform: "(1) Anæsthesia is obtained in a very short time, two minutes at the outside, and without a preliminary stage of excitement. (2) A dose of five grammes (seventy-five minims) of bromide of ethyl is sufficient on an average to produce complete anæsthesia. (3) The administration of this substance is unattended by alarming manifestations or accidents of any kind. (4) The quantity of chloroform administered is much less with the mixed than with the ordinary method, seeing that with the former the dose employed seldom exceeds thirty grammes (one ounce). The administration of seventy centigrammes (ten minims) per minute is usually sufficient to keep up the anæsthesia throughout the operation. (5) Lastly, secondary manifestations due to the action of the anæsthetic are never observed after an operation performed under bromethyl-chloroform anæsthesia."

Pavlow also states that "nausea and vomiting are very rare with this anæsthetic (bromethyl), which may be employed in even the most delicate operations."

Professor Pawloff[32] arrives on almost precisely similar conclusions to Professor Pavlow.

Magill[33] strongly advocates the use of the mixed anæsthetics, chloroform and ethyl-bromide. He says, "the dangers attributed to the use of chloroform alone are in a great measure eliminated by the counter action of ethyl-bromide previously administered." He agrees with the other advantages described above, and quotes Tenier's method of administration. Haltman and Bourbon[34] make allusion to the fact that in some cases after the administration of ethyl-bromide the patient's breath may smell for a day or two of garlic, but of this he is not conscious. Legond[35] has had a large experience in the administration of bromethyl and bromethyl-chloroform. He states that "never in a single case has he met with the slightest accident, neither in the course of administering the bromethyl nor during the period when this substance had given place to chloroform." He says he has never observed any vomiting from the use of bromethyl alone, and describes the method adopted. Bazy has observed vomiting with bromethyl however, and Monod in the same paper says that in the first moments of administration of bromethyl the anxiety and the sensation of suffocation are so great that he prefers chloroform alone. Berger at the same discussion said that in Prof. Goselin's ward he was struck with the brutal and painful manner in which anæthesia is produced with this drug, and with the unpleasant recollection the patients generally retain of this circumstance. This was the principal reason of his giving up its use. Lucas-Championnière refers to violent agitation as always being present, and says it seemed to him "that after producing anæsthesia by means of bromethyl just as much chloroform had to be administered as if no bromethyl at all had been given. In my opinion the successive use of bromethyl and chloroform is a useless complication, and gives no better results than those obtained by chloroform alone when one knows how to employ the latter."

Monod[36] "insists on the repugnance shown by patients to bromethyl as well as on the sometimes very disquieting symptoms which mark the beginning of the anæsthesia, and which in two cases out of fifty made artificial respiration a necessity. With regard to vomiting, this complication was only absent twice out of fifty cases, and in ten the vomiting continued for over three days."

Kochler[37] describes a death during the administration of ethyl-bromide. A weak but otherwise healthy woman at twenty-one was about to be operated on for rectal fistula. The anæsthetic was given in small doses on a mask. After a transient and mild stage of excitement the heart's action suddenly ceased. The breathing continued for half an hour, but, notwithstanding subcutaneous injections of ether and intravenous injection of saline solution, the patient never rallied. The author considers the fatal result as due to cardiac paralysis. The autopsy showed extreme fatty degeneration of the muscular structure of the heart. Reference is made to five recorded deaths under this anæsthetic. Acording to Gurlt's statistics the proportion of deaths is one in 4,555.

**Pental.**—Phillip,[38] after a year's experience of this anæsthetic, ascribes the following advantages to it:—Extraordinarily rapid narcosis, rarity of a period of excitement which, if present, ceased with absolute narcosis; immediate recovery of consciousness after removal of the mask; and absence of any unpleasant after effects such as are caused by chloroform. No action on the heart was ever observed, but in some patients arrest of respiration and cyanosis occasionally appeared. This cyanosis is attributed to tonic contractions of the diaphragm and glottis. The author, in conclusion, judging by 1,000 narcoses, considers that pental, with certain exceptions, is always able to replace chloroform, being, moreover, less dangerous and without after effects. All precautions should be taken during its use as in the administration of chloroform. Dr. David Cerna[39] in dealing with this subject agrees that anæsthesia is rapidly established, but says it also quickly disappears; that pental depresses the circulation to a dangerous degree, causing a fall of the arterial pressure and of the rate of the pulse; that the respiratory rate is increased through a direct action of the agent upon the respiratory centres; that sometimes Cheyne-Stokes' respiration is produced; that it causes death by cardiac paralysis, sometimes, however, heart and respiration stop simultaneously; at others a fatal issue is the result of respiratory failure; that the narcosis is not unattended by unpleasant after effects, the action of these being principally that of excitement; that it cannot be considered as a safe or even as an efficient general anæsthetic, and is certainly inferior to ether and chloroform. The latter observations will be borne out by Gurlt's statistics, in which he finds the very high mortality of three deaths in 597 cases of pental narcosis. The other death dates collected by this observer, not previously mentioned, are one in 4,118 cases of mixed chloroform and ether narcosis, and there were 3,440 cases in which the A.C.E. mixture was used without a death. As a local anæsthetic, Gibson[40] recommends ethyl chloride; he describes its properties and applicability.

[18] Med. Week, March 9th. [19] N. Y. Med. Jour., June 23rd. [20] Med. Week, Feb. 9th. [21] Centralblatt für Chirurgie, Jan. 20th. [22] Amer. Jour. Med. Sci., Feb., p. 192. [23] Med. Week, March 9th. [24] Amer. Jour., March, p. 332. [25] B. M. J. Epit., March 31st. [26] Lancet, March 24th. [27] Lancet, March 31st. [28] Lancet, April 7th. [29] Ther. Gaz., Feb., p. 115. [30] N. Y. Med. Jour., May 26th. [31] Med. Week, Feb. 9th. [32] Ther. Gaz., May 15th. [33] Med. Reprints, Feb. 15th. [34] Amer. Journ. Med. Sci., Feb., p. 211. [35] Med. Week, May 11th. [36] Med. Week, May 25th. [37] B. M. J. Epit., March 10th. [38] Ibid., Feb. 17th. [39] Amer. Jour. Med. Sci., Feb., p. 193. [40] Ther. Gaz., Feb. 15th.

